# Contour Fillings—When Are They Indicated?

**Published:** 1869-07

**Authors:** H. L. Sage

**Affiliations:** Bridgeport, Conn.


### CONTOUR FILLINGS—WHEN ARE THEY INDICATED?

BY H. L. SAGE, BRIDGEPORT, CONN.

Although some diversity of opinion may exist relative to the *when* and *where*, in considering the indications of contour filling, I trust no apology is necessary for presenting a brief *resumé* of the cases in which such indications exist. In coming to a conclusion much judgment may be required in what may, at first sight, appear a plain case. Whether or not we are, in some cases, to shock the good taste of nature-worshipers, in order to avoid a greater and more serious calamity. The aim in the construction of the artificial seems to be to counterfeit nature so closely as to place its false character beyond detection, at least by the mere casual glance of the eye. Hence, the objection that obtains in the minds of many to any thing that falls short of such a standard, and hence the objections to contour fillings. Health, comfort and permanent good are often sacrificed to this false pride; so much so, that many prefer to lose those valuable and useful organs, the teeth, in order that they may be replaced by artificial substitutes *in full*, that do not show to the unskilled eye their true character, rather than to have a portion of the lost structure replaced in a more useful and enduring way, because it shows an infringement of the pleasing in nature. Do these patients ever consider what they would be if deprived of their gold and porcelain? "Between two evils choose the least," though the greater may not be so apparent as it really is. Every honest Dentist knows how hard it is at times to persuade a patient not to sacrifice his or her teeth to the "tender mercies" of the mere mechanical manipulator. And if he lives up to his duty and refuses to compromise his convictions in this respect, he must not unfrequently expect to exchange a prospective pecuniary reward, for self respect, and "a conscience void of offense." The rule should be, conceal the artificial character of your operations as much as possible, but not at the expense of the future welfare of the organs you attempt to save.

But to revert to the question under consideration, I would suggest that contour fillings are indicated, first, when the borders of the cavity are so frail that there is danger of fracturing the enamel in putting in the filling, notwithstanding requisite care be used, or there would be a probability of its breaking away from the filling after its insertion, by the ordinary mastication of the food. In such a case it is much the best plan to cut away the frail edges and restore to the original shape with gold, *i. e.*, sacrifice beauty to strength and durability, and in the long run to comfort. When the extent of the cavity does not necessitate the cutting off the approximal surface at the point, but it takes on the usual semi-circular shape, and the labial and lingual walls are very thin—so much so as to make it unsafe to leave them, owing to the dangers above stated—in that case it is best to cut away with a fine, half-round file, until strong borders are secured and then restore with gold.

Second—Contour fillings are indicated when, in the case of the incisors or canines, much space is left between them by the decay of the approximal surfaces, thus interfering with speech or destroying the natural symmetry of the teeth.

Third—In the case of the bicuspids and molars, when much of the tooth structure is broken away or the borders of the cavity are frail, and it is desirable to obtain as much grinding surface as was had in the original shape of the tooth.

Fourth—In almost all cases when the dentine would be exposed to the chemical action of the fluids of the mouth, thus inducing decay; and if it is admissible to leave it exposed at all, which is questionable in the majority of cases, the front teeth would constitute the exception. Generally speaking, the borders of the cavity, when the enamel has been cut away and the dentine exposed, (which should never be done unnecessarily) should be protected by building out and lapping over, more or less, according to the requirements of the case. If left without protection, as in the case of the front teeth, the dentine should be highly polished by the usual methods.

When are contour fillings *partially* indicated?

First—When, in the case of teeth very much crowded, it would not be necessary to build out the tooth to its original shape fully, but only enough to serve as a protection to the dentine, though much of the structure is lost.

Second—In cases of grinding teeth when three fourths, say, of the crown is gone, one approximal surface, for instance, being perfect, and it being very difficult, by reason of the close proximity of caries to the nerve, to obtain sufficient retaining points to render a large filling firm in its attachments. Then build it out no more than is compatible with safety or strength, for the more surface you expose to friction, in such a case, the more liable would the filling be to loosen or fall out.

It is my practice to make a square cut across the frail border, from the labial to the lingual surface, semi-circular or straight, as the case may require, leaving the edges quite smooth, but not beveled, so that when the gold is built against these edges it may present a joint with the tooth as perfect as it would be possible to unite two perfectly plain and smooth pieces of wood, and more so, impervious to the fluids of the mouth, and this idea should be carried out, beginning with the cervical portion and extending to every part of the cavity, the connection being perfect inside and out. No projection of gold should be allowed beyond the enamel, but it should be flush and plump with it.

On grinding teeth, it should not be so full as to prevent the sound ones from antagonizing properly, and if there is to be any difference in the amount of force of occlusion on the distributing surface, the teeth without fillings should receive the greatest, as it is always unpleasant to the patient to have the filling seem too long in biting upon it, to say nothing of the injury produced to both teeth and filling. By the above mode of practice, briefly stated, my success has not been barren of good results. Perhaps others may suggest better methods, or improvements upon the foregoing.

